# Short report: Twins with 20p13 duplication. Case report and comprehensive literature review

**DOI:** 10.1002/mgg3.2436

**Published:** 2024-05-13

**Authors:** Benjamin J. Kennedy, Sarah K. Savage, Stephen G. Kaler

**Affiliations:** ^1^ Center for Gene Therapy, Abigail Wexner Research Institute Nationwide Children's Hospital Columbus Ohio USA; ^2^ Division of Genetic and Genomic Medicine Nationwide Children's Hospital Columbus Ohio USA

**Keywords:** 20p13 duplication, gene duplication, syntaphilin, trisomy 20p, twin‐twin transfusion syndrome

## Abstract

**Background:**

Trisomy 20p is a rare genetic condition caused by a duplication of the short arm of chromosome 20.

**Methods:**

We employed clinical observation and molecular genetic testing (SNP microarray), to study identical twin males with an unknown dysmorphic syndrome. We conducted a literature review of trisomy 20p and collated the clinical and molecular genetic findings on 20 affected subjects reported since 2000.

**Results:**

Identical twin males, whose prenatal course was complicated by a twin‐to‐twin transfusion, manifested profound language and neurocognitive delays as well as distinctive facial dysmorphisms when evaluated at 2 years of age. SNP microarray identified identical duplications of 20p13 with no other chromosomal aberrations. A literature survey of 20p trisomy syndrome identified 20 other examples of this condition reported since 2000, which we collated with 33 summarized by Sidwell et al. (2000). Within the combined total of 55 affected individuals, we found a distinctive clinical phenotype that provides insight on the effects of abnormal dosage of genes in 20p13. These loci include *FAM110A* (OMIM 611393), *ANGPT4* (OMIM 603705), *RSPO4* (OMIM 610573), *PSMF1* (OMIM 617858), *SNPH* (OMIM 604942), *SDCBP2* (OMIM 617358), *FKBP1A* (OMIM 186945), *TMEM74B*, *C20orf202*, and *RAD21L1* (OMIM 619533). Gene profiling highlighted that syntaphilin (*SNPH*) is highly expressed in mammalian brain, where it is considered critical for mitochondrial transport in neuronal axons, and to directly influence axonal morphogenesis and function.

**Conclusion:**

We propose that abnormal activity of syntaphilin engendered by the trisomy is primarily responsible for the language, neurocognitive, and gross motor delays reported in individuals with 20p trisomy. Additional studies, for example, characterization of cerebral organoids generated from affected patients may help to better understand this condition, and potentially suggest rational remedies to improve the lives of affected individuals and their families.

## Graphical abstract

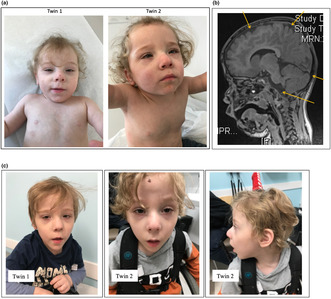

## 1 INTRODUCTION

Trisomy 20p is a rare genetic condition caused by a duplication of the short arm of chromosome 20 (de Ravel et al., 2003). Duplications of the short arm of chromosome 20 can vary in size; some span the entire arm while others include just a fraction, which may contribute to phenotypic variation among patients (Khattak et al., 2020; Oppenheimer et al., 2000). A defined phenotype for the condition remains equivocal as trisomy 20p is commonly reported in association with monosomy of the same or of another chromosome, and the extent of the duplication is variable (Chaabouni et al., 2007; de Ravel et al., 2003). Cases of trisomy 20p occurring with no other chromosomal aberrations are uncommon (Bartolini et al., 2013; Choi et al., 2020; Sidwell et al., 2000). Trisomy 20p is most frequently the result of parental reciprocal translocation, while de novo cases rarely emerge (Bartolini et al., 2013; Chaabouni et al., 2007; Choi et al., 2020; Khattak et al., 2020; Sidwell et al., 2000). Sidwell et al. (2000) advanced understanding of the phenotypes associated with this condition by describing a case of de novo pure trisomy 20p, along with 32 previously reported cases (Sidwell et al., 2000). Here, we describe trisomy 20p in twin males with identical duplications of 20p13 without any other known chromosomal aberrations, a genetic anomaly not previously described or associated with a defined phenotype. We also provide an updated review of literature relevant to this topic from the past 20 years (Table 1).

**TABLE 1:** Clinical features of affected twins and 20 cases (Bartolini et al., 2013; Batanian et al., 2014; Chaabouni et al., 2007; Choi et al., 2020; de Ravel et al., 2003; Della‐Rosa & Vianna‐Morgante, 2000; DeScipio et al., 2010; Kang et al., 2012; Khattak et al., 2020; Kwon et al., 2018; Leclercq et al., 2009; Oppenheimer et al., 2000; Pachajoa et al., 2020; Sidwell et al., 2000; Thomas et al., 2003; Trachoo et al., 2013; Une et al., 2006; Venditti et al., 2003; Wieczorek et al., 2003) of trisomy 20p reported since 2000.

Case	Case#	Gender	Gestation (weeks)	Birth weight (g)	Developmental delay	Speech delay	Cardiac anomalies	Digital anomalies	Dysmorphic facial features	Oral facial anomalies	Hair anomalies	Vertebral anomalies
Present case	1	M	25	832	+	+	+	+	+	−	+	−
	2	M	25	611	+	+	−	+	+	−	+	−
Sidwell (2000)	3	M	21	2500	+	+	−	−	+	+	+	+
Oppenheimer (2000)	4	M	Term	2200	+	+	+	+	+	+	+	+
	5	F	41	3000	+	+	−	+	+	+	+	+
Della‐Rosa (2000)	6	F	Post‐Term	2450	+	+	NR	+	+	+	NR	+
de Ravel (2003)	7	F	Term	2790	+	+	−	−	+	−	−	NA
Wieczorek (2003)	8	M	34	2510	+	NR	+	+	+	+	+	NR
Thomas (2003)	9	M	39	2335	NR	NR	+	+	+	−	NR	NR
Venditti (2003)	10	M	37	2500	+	NA	+	+	+	+	NR	NR
Une (2006)	11	F	40	3949	+	NR	+	NR	+	+	+	NR
Chaabouni (2007)	12	M	37	3450	+	+	+	+	+	+	+	+
Leclercq (2009)	13	M	36	2880	+	+	−	+	+	+	+	NA
DeScipio (2010)	14	M	36	2330	+	+	+	−	+	+	NR	NR
Kang (2012)	15	F	38 + 2	2350	+	+	+	+	−	NR	NR	NR
Bartolini (2013)	16	M	36	3100	+	+	−	+	+	−	+	+
Batanian (2014)	17	M	NR	NR	+	+	+	+	+	NR	+	NR
Trachoo (2013)	18	F	38	2900	+	+	NR	−	+	+	−	NR
Kwon (2018)	19	F	34	3840	+	+	NR	NR	+	NR	NR	NR
Khattak (2020)	20	F	37	2551	+	+	+	+	+	+	−	−
Choi (2020)	21	F	NR	NR	+	+	−	−	+	NR	NR	−
Pachajoa (2020)	22	F	NR	6000	+	+	−	+	+	−	−	−
Summary	*n* = 22	12M/10F	21–41	611–6000	100%	100%	58%	75%	95%	67%	73%	55%
Sidwell Table 1 (2000)	*n* = 32	16M/16F	21–25	1800‐4300	94%	95%	36%	92%	62%*	71%**	78%	81%
Combined Total	*n* = 54	28M/26F	21–41	611–6000	96%	98%	45%	82%	62%–95%	67%–71%	76%	72%

Abbreviations: NA, not assessed; NR, not reported; +, present; −, absent.

* Mean percentage of the following facial features reported by Sidwell (epicanthus, strabismus, increased inner canthal distance, upslanting palpebral fissures, round face/prominent cheeks, short upturned nose, large flared nostrils, large or abnormal ears, moderate micrognathia).

** Mean percentage of the following clinical features reported by Sidwell (high‐arched or cleft palate, dental anomalies).
Trisomy 20p with karyotype: 46,XY,der(4)t(4:20) (pter;q11.1),i(20)(q11.1). Partial trisomy (20)(pter, p11.1). Partial trisomy 20p due to Dup20pter → 20q12; Del14pter → 14q11.1. Partial trisomy 20p due to tandem duplication: 20p12.1 → p13. Partial monosomy 18p; partial trisomy 20p due to unbalanced translocation: 46,XY,der18 t(18;20)(p11.2;p12.3)pat. Partial monosomy 17p; partial trisomy 20p due to unbalanced translocation: 46,XY,der17 t(17;20)(p13.3;p12.2)mat. Mosaic trisomy 20p due to 46XY, pseudodicentric (20;20)(p13;p13),+20. Trisomy 20p: 46,XX,dup (20)(p11.2p13). Trisomy 20p: 46,XY,der(20)(pter → q13.3::p11.2 → pter). Partial trisomy 20p due to duplication from 20p13 to 20p11.22. Concurrent terminal deletion, 20p13 region. Partial trisomy 20p, partial monosomy 20q due to mosaic maternal pericentric inversion. Partial trisomy 20p due to meiotic recombination of a maternal pericentric inversion, rec(20)dup(20p). Trisomy 20p due to de novo duplication of 20p: 46,XY,dup(20)(p11.2p13).Concurrent small duplication of 3p. Partial trisomy 20p due to duplication of 20p12.2–11.2; concurrent duplication of 16p11.2. Partial trisomy 20p due to duplication of 20p11.22‐20p13; concurrent deletion of 20p13‐20pter. Partial trisomy 20p due to duplication of 20p13‐p12.3; concurrent small 20p13 deletion. Partial trisomy 20p due to duplication of 20p12.2. Trisomy 20p due to duplication of 20p13‐p11.1. Partial trisomy 20p due to duplication of 20p13p12.1.

## 2 METHODS

### 2.1 Ethical compliance

Signed informed consent for publication of de‐identified medical information and photographs was obtained from the patients' legal guardian.

## 3 CASE REPORTS

Patients 1 and 2 are 5‐year‐old male monochorionic, diamniotic twins who presented with dysmorphic facial features, developmental delays, and minor toe anomalies. They were born by spontaneous vaginal delivery at 25 weeks gestation to a 29‐year‐old mother and 25‐year‐old father. Their birth was complicated by premature rupture of membranes and onset of labor, and twin‐to‐twin transfusion syndrome (TTTS), resulting in unequal birthweights. The birthweight of Twin 1 (TTTS recipient) was 832 g, while the birthweight of Twin 2 (TTTS donor) was 611 g.

The family history includes cerebral palsy, hydrocephalus, developmental delay, and stroke in their natural mother; asthma and sleep apnea in their natural father; cancer, heart disease, and asthma in their paternal grandmother; asthma in their maternal grandfather; and autism in another family member.

**FIGURE 1:**
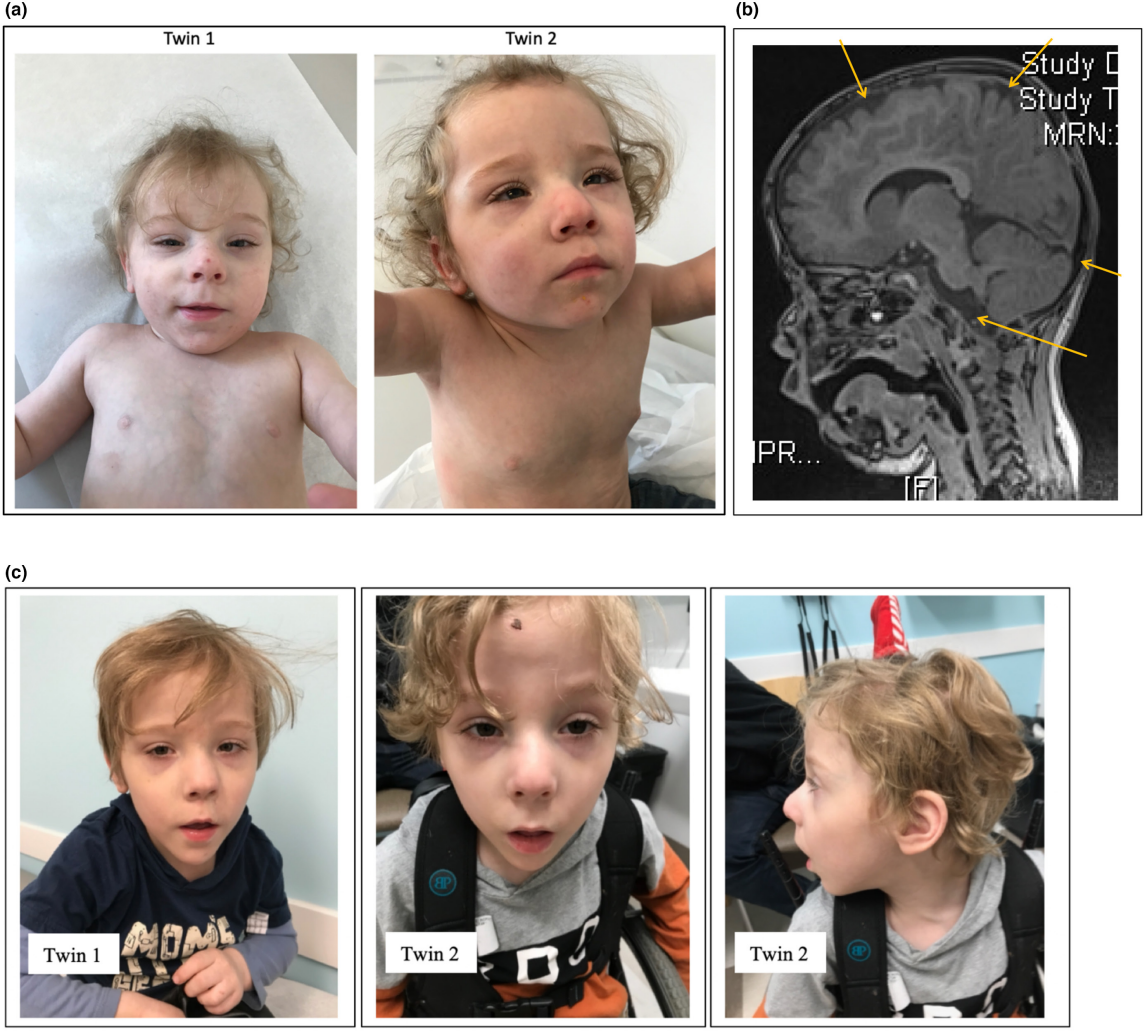
(a). Facial dysmorphism in twins with 20p13 duplication. Note thin hair, deep‐set eyes, hypertelorism, narrow palpebral fissures, long eyelashes, broad nasal bridge; bulbous nose, and long philtrum. Age: 2 years, 2 months. (b) T1‐weighted midline sagittal image in Twin 1. Mild nonspecific prominence of supratentorial sulci and cisterns consistent with mild cerebral volume loss (arrows). Minimal increased FLAIR signal consistent with periventricular gliosis or leukomalacia. (c) Dysmorphic facial features in twins with 20p13 duplication syndrome, at 5 years of age. Note triangular facies, in comparison to younger age.

The twins presented with global developmental delays; of which language and personal‐social spheres were more affected than gross motor and fine motor. Both manifested tremors and low truncal tone. They showed normal somatic growth corrected for prematurity. Clinical examination revealed dysmorphic facial features in both twins, including hypertelorism, small upturned and deep‐set eyes, narrow palpebral fissures, long eyelashes, broad nasal bridge, bulbous nose, and a long philtrum (Figure 1a).

Thin hair, broad chests with wide‐spaced nipples (11.5 cm; >95th centile for age), slightly overlapping toes (#3 over #2), superficial venous patterns on the thorax, and small umbilical herniae were also observed. Both had a history of bronchopulmonary dysplasia, and Twin 1 was noted with a patent foramen ovale, which resolved. Brain imaging disclosed mild, nonspecific prominence of supratentorial sulci and cisterns in both twins, consistent with possible mild cerebral volume loss in the setting of prematurity. Minimal increased FLAIR signal was observed in Twin 1, consistent with periventricular gliosis/leukomalacia (Figure 1b).

At 5 years of age, the twins have made considerable developmental progress. Twin 1 sits, crawls, stands and walks independently, although with some ataxia. Twin 2 sits independently, and crawls but does not walk independently. They have both been diagnosed as having level 3 autism spectrum disorder.

Their hair remains thin in texture and their faces are now distinctively triangular (Figure 1c) in addition to the features noted in infancy (epicanthal folds, hypertelorism, bulbous nose, micrognathia, long philtrum, and large, low‐set ears).

## 4 GENETIC ANALYSIS

Agilent GGXChip+SNP v1.0, which features approximately 120,000 CGH probes and 60,000 SNP probes, indicated a 572.21 kb gain within chromosome band 20p13 in both twins: arr[GRCh37] 20p13(824997_1397204)x3. This 20p13 gain includes the 5′ untranslated region of *FAM110A*, along with the coding regions of nine additional genes: *ANGPT4*, *RSPO4*, *PSMF1*, *SNPH*, *SDCBP2*, *FKBP1A*, *TMEM74B*, *C20orf202*, *RAD21L1*, (Table 2), 3 long non‐coding RNAs (*LOC105372493*, *FKBP1A‐SDCBP2*, *SDCBP2‐AS1*), and 1 microRNA (*MIR6869*). No region of monosomy was detected.

**TABLE 2:** Relevant genes and gene products with known functions involved in 20p13 duplication.

Gene/Reference Seq	Product	OMIM	Function
*FAM110A* NM_001042353.3	FAM110A	611393	Localized in cytoplasm and predicted to serve as a microtubule organizing center and spindle pole. Highly expressed in the skin and spleen
*ANGPT4* NM_015985.4	Angiopoietin‐4	603705	Serves important roles in vascular development and angiogenesis. Highly expressed in fatty tissue
*RSPO4* NC_000020.11 (958452..1002311, complement)	R‐spondin 4	610573	May be involved in Wnt/beta‐catenin signaling pathways. Highly expressed in the lungs and testes
*PSMF1* NC_000020.11 (1113263..1172246)	Proteasome inhibitor Subunit 1	617858	Inhibits the 11S and 19S regulators of the 26S proteasome, a multi‐catalytic proteinase complex
*SNPH* NC_000020.11 (1266294..1309327)	Syntaphilin	604942	Axonally‐targeted static anchor protein that immobilizes mitochondria in neuronal axons. Directly associates with mitochondria and microtubules to anchor the former, thereby controlling mitochondrial transport for axonal morphogenesis and function. Highly expressed in the brain
*SDCBP2* NC_000020.11 (1309909..1329139, complement)	Syndecan binding protein 2	617358	Serve critical roles in cell signaling and protein complex organization
*FKBP1A* NC_000020.11 (1368978..1393054, complement)	FKBP propyl‐isomerase 1A	186945	Serves roles in immunoregulation as well as protein folding and trafficking
*TMEM74B* NC_000020.11 (1180570..1189409, complement)	Transmembrane protein 74B	Not listed	Critical component of the cell membrane. Broadly expressed, especially high in the small intestine, placenta, duodenum, and the lungs
*C20orf202* NC_000020.11 (1203454..1209076)	Chromosome 20 open reading frame 202	Not listed	Function(s) unknown
*RAD21L1* NC_000020.11 (1226044..1255876)	RD21L	619533	Enables chromatin binding and mitotic sister chromatid cohesion. Restricted expression to the testes

## 5 LITERATURE REVIEW AND DISCUSSION

Clinical features of patients harboring chromosome 20p duplications have previously been described, and common phenotypic features among 33 cases identified up to 2000 (Sidwell et al., 2000). We compared the clinical features of our two patients to those findings and to those of 20 additional cases involving trisomy 20p reported since 2000 (Table 1). Very few cases are reported as pure trisomy 20p, in which no other chromosomal material is involved (Choi et al., 2020; Sidwell et al., 2000). Most cases were reported as partial trisomy associated with distal monosomy of the same or another chromosome (Chaabouni et al., 2007; Della‐Rosa & Vianna‐Morgante, 2000; DeScipio et al., 2010; Kang et al., 2012; Kwon et al., 2018; Leclercq et al., 2009; Thomas et al., 2003; Trachoo et al., 2013; Wieczorek et al., 2003). The majority of cases emerged from parental reciprocal translocation, while fewer emerge de novo (Bartolini et al., 2013; Chaabouni et al., 2007; Choi et al., 2020; Khattak et al., 2020; Sidwell et al., 2000).

The 22 total cases considered in this investigation reflect common clinical features in the following categories: developmental delay, speech delay, cardiac anomalies, digital anomalies, dysmorphic facial features, oral facial anomalies, hair anomalies, and vertebral anomalies. Developmental delay was reported in many ways including global developmental delay, psychomotor delay, fine and gross motor delay, and poor coordination. Any combination of which was recorded as developmental delay in this study. Developmental delay was reported in 100% of the cases we investigated, including our own, and 94% of the cases investigated by Sidwell et al. (2000). The most frequently reported delayed developmental milestones included motor achievements, such as walking, as observed in our patients (Chaabouni et al., 2007; Della‐Rosa & Vianna‐Morgante, 2000; DeScipio et al., 2010; Kang et al., 2012; Khattak et al., 2020; Leclercq et al., 2009; Oppenheimer et al., 2000; Sidwell et al., 2000). Speech and/or language delays were reported in 100% of our cases and in 95% of those investigated by Sidwell et al. (2000). In our patients, speech is limited to irregular vocalizations. At age 5 years, neither of them speak in word combinations.

Of literature cases since 2000, 58% reported cardiac anomalies while Sidwell et al. recorded cardiac anomalies in 36% of the cases they reviewed (Sidwell et al., 2000). Cardiac anomalies reported in the cases we investigated included: ventricular septal defect (Batanian et al., 2014; Kang et al., 2012; Oppenheimer et al., 2000; Une et al., 2006), tetralogy of fallot (Batanian et al., 2014; Wieczorek et al., 2003), double outlet right ventricle (Batanian et al., 2014; Thomas et al., 2003), Dextro‐transposition of the great arteries (Thomas et al., 2003), pulmonary atresia (Kang et al., 2012; Thomas et al., 2003), small pulmonary arteries (Thomas et al., 2003), patent ductus arteriosus (Kang et al., 2012; Thomas et al., 2003; Venditti et al., 2003), heart murmur (DeScipio et al., 2010; Kang et al., 2012; Thomas et al., 2003), dysplastic mitral valve (Venditti et al., 2003), aortic valvular stenosis (Venditti et al., 2003), and atrial septal defect (Batanian et al., 2014; Khattak et al., 2020).

A definitive association between a specific cardiac anomaly and trisomy 20p remains indeterminant. Patent foramen ovale was observed in Twin 1, but no persistent cardiac murmur was found in our patients. Digital anomalies were found in 92% of the cases reviewed by Sidwell et al. (2000), and in 75% of the cases we investigated, including our own. These included clinodactyly (Kang et al., 2012; Khattak et al., 2020; Leclercq et al., 2009; Oppenheimer et al., 2000), brachydactyly (Chaabouni et al., 2007), broad distal phalanges of thumbs/toes (Della‐Rosa & Vianna‐Morgante, 2000), toes of unequal length and overlapping toes (Thomas et al., 2003). Along with developmental delays, dysmorphic facial features have frequently emerged in association with trisomy 20p, occurring in 95% of the cases we investigated, including our own, and 62% of the cases reviewed by Sidwell et al. (2000). Commonly reported dysmorphic facial features in the setting of trisomy 20p include round face with prominent cheeks (Bartolini et al., 2013; Batanian et al., 2014; Chaabouni et al., 2007; de Ravel et al., 2003; Della‐Rosa & Vianna‐Morgante, 2000; Khattak et al., 2020; Leclercq et al., 2009; Oppenheimer et al., 2000; Pachajoa et al., 2020; Trachoo et al., 2013; Une et al., 2006; Wieczorek et al., 2003), broad nasal bridge (Bartolini et al., 2013; Della‐Rosa & Vianna‐Morgante, 2000; Oppenheimer et al., 2000; Pachajoa et al., 2020; Thomas et al., 2003; Trachoo et al., 2013), anteverted nares (Bartolini et al., 2013; Chaabouni et al., 2007; Khattak et al., 2020; Thomas et al., 2003; Une et al., 2006), short nose (Bartolini et al., 2013; Khattak et al., 2020; Leclercq et al., 2009; Trachoo et al., 2013; Wieczorek et al., 2003), micrognathia (Bartolini et al., 2013; Chaabouni et al., 2007; Choi et al., 2020; Khattak et al., 2020; Sidwell et al., 2000; Thomas et al., 2003; Venditti et al., 2003), hypertelorism (Oppenheimer et al., 2000; Une et al., 2006; Wieczorek et al., 2003), abnormal palpebral fissures (narrow, up slanted, down slanted) (Bartolini et al., 2013; Batanian et al., 2014; Chaabouni et al., 2007; Della‐Rosa & Vianna‐Morgante, 2000; Khattak et al., 2020; Oppenheimer et al., 2000; Pachajoa et al., 2020; Sidwell et al., 2000; Thomas et al., 2003; Trachoo et al., 2013; Une et al., 2006), abnormal philtrum (short, long, featureless) (Bartolini et al., 2013; Chaabouni et al., 2007; Khattak et al., 2020; Venditti et al., 2003), epicanthus(Bartolini et al., 2013; Khattak et al., 2020; Leclercq et al., 2009; Pachajoa et al., 2020; Sidwell et al., 2000; Thomas et al., 2003; Trachoo et al., 2013) and strabismus (Bartolini et al., 2013; Choi et al., 2020; Della‐Rosa & Vianna‐Morgante, 2000; Khattak et al., 2020). Of these features, broad nasal bridge, hypertelorism, abnormal palpebral fissures, and philtrum abnormalities were apparent in our twin patients. Oral facial anomalies, such as high arched palate (Chaabouni et al., 2007; Della‐Rosa & Vianna‐Morgante, 2000; Leclercq et al., 2009; Oppenheimer et al., 2000; Sidwell et al., 2000), small teeth (Chaabouni et al., 2007), wide spaced teeth (Wieczorek et al., 2003), thin‐upper lip (Della‐Rosa & Vianna‐Morgante, 2000; DeScipio et al., 2010), and other dental anomalies (Batanian et al., 2014; Oppenheimer et al., 2000; Trachoo et al., 2013; Une et al., 2006) were also reported in 67% of the cases we investigated, and 71% of the cases reviewed by Sidwell et al., but were not observed in our patients (Sidwell et al., 2000). Anomalies of the hair, commonly reported as coarse (Bartolini et al., 2013; Leclercq et al., 2009; Oppenheimer et al., 2000; Sidwell et al., 2000; Une et al., 2006; Wieczorek et al., 2003) or thin hair, occurred in 73% of our cases, including our own, and 78% of the cases reviewed by Sidwell et al. (2000).

Vertebral anomalies, while found to be well represented in the cases reviewed by Sidwell et al. (2000), have been less represented since, and are absent in our patients. Vertebral anomalies in the setting of trisomy 20p were reported in 55% of the cases we investigated. Kyphosis has been reported most frequently (Bartolini et al., 2013; Chaabouni et al., 2007; Della‐Rosa & Vianna‐Morgante, 2000; Oppenheimer et al., 2000) while fused and collapsed vertebrae have been reported as well (Oppenheimer et al., 2000; Sidwell et al., 2000).

Other phenotypic anomalies reported in association with trisomy 20p include wide spaced nipples (Chaabouni et al., 2007; de Ravel et al., 2003; DeScipio et al., 2010; Wieczorek et al., 2003), long eyelashes (Bartolini et al., 2013; DeScipio et al., 2010) and deep‐set eyes (Venditti et al., 2003), each of which were observed in our patients. Prominent veins on the abdomen were noted by Thomas et al. (2003), reminiscent of the superficial venous pattern observed on our patients. Renal anomalies such as small kidney (Sidwell et al., 2000), absent left/right kidney (Oppenheimer et al., 2000; Trachoo et al., 2013), renal insufficiency (Wieczorek et al., 2003), bilateral renal dysplasia (Wieczorek et al., 2003), chronic parenchymatous disease (Trachoo et al., 2013), and hyperechoic kidneys (Thomas et al., 2003) have been previously reported, but were not observed in our patients. Normal growth is commonly associated with trisomy 20p (Bartolini et al., 2013; Chaabouni et al., 2007; Leclercq et al., 2009; Sidwell et al., 2000), consistent with that of our patients.

We conclude that trisomy 20p13 is an uncommon chromosomal copy number variant associated with a distinctive facial appearance and variable neurodevelopmental and neurocognitive manifestations. Of the 10 genetic loci involved in the duplication, syntaphilin (*SNPH*) appears potentially most relevant to the clinical phenotype. This gene is highly expressed in mammalian brain, where its protein product is considered essential for mitochondrial transport in neuronal axons and to influence axonal morphogenesis and function (Lao et al., 2000). Proper distribution of mitochondria within axons and at synapses is critical for neuronal function. Axonal mitochondria that contain exogenously or endogenously expressed SNPH lose mobility, permitting maintenance of normal regional axonal mitochondrial density. In contrast, deletion of the mouse homolog, *snph*, results in a substantially higher proportion of axonal mitochondria in the mobile state, reducing mitochondrial density in axons (Kang et al., 2008). We speculate that the normal control of axonal mitochondrial mobility is perturbed in subjects with an additional copy of SNPH, due to 20p13 duplication. Additional research is needed to formally evaluate this hypothesis. Generation of pluripotent stem cells, and subsequently cerebral organoids, from cultured fibroblasts of these patients may enable fruitful characterization of the genetic effects. Clinical follow‐up of the twins reported here and other affected individuals will also further enhance understanding of the natural history and long‐term prognosis of this rare gene duplication syndrome.

## AUTHOR CONTRIBUTIONS

BJK performed the literature review and wrote the initial draft of the manuscript. SKS evaluated the patients and reviewed serial drafts of the manuscript. SGK examined the patients, wrote and edited the manuscript, and reviewed the literature.

## FUNDING INFORMATION

Abigail Wexner Research Institute (40301‐0008).

## CONFLICT OF INTEREST STATEMENT

None of the authors have conflicts of interest to disclose.

## ETHICS STATEMENT

We obtained approval and signed consent for publication of de‐identified medical information and photographs from the twins' parent/legal guardian. IRB approval of single case report data is not a requirement at Nationwide Children's Hospital, as per the NCH Investigator Manual: “For a case report (1‐2 cases), an investigator does not need prior IRB review and approval if: (a) the records accessed are available to the investigator for clinical reasons (i.e., they or direct colleagues were involved in the care of this patient), (b) the records being reviewed contain data that were collected as part of routine clinical care, and (c) the data are reviewed in a retrospective manner.”

## Data Availability

The data that support the findings of this study are available from the corresponding author upon reasonable request.
